# Masticatory and Neck Muscles’ Isometric Endurance and Their Relation to Upper Limb Isometric Grip Strength in Sport Climbers—Preliminary Investigation

**DOI:** 10.3390/medicina60121956

**Published:** 2024-11-27

**Authors:** Michał Baszczowski, Aleksandra Dolina, Magdalena Zawadka, Michał Ginszt, Adam Czarnecki, Agata Ginszt, Piotr Gawda

**Affiliations:** 1 Department of Sports Medicine, Medical University of Lublin, 20-093 Lublin, Poland; 2 Department of Rehabilitation and Physiotherapy, Medical University of Lublin, 20-093 Lublin, Poland; 3 Interdisciplinary Scientific Group of Sports Medicine, Department of Sports Medicine, Medical University of Lublin, 20-093 Lublin, Poland

**Keywords:** sEMG, sport climbing, isometric manual grip strength, isometric endurance, masticatory muscle, neck muscle

## Abstract

*Background and Objectives*: Sport climbing’s popularity has grown with its inclusion in the Olympics and increased accessibility. Understanding the relationship between hand dominance, grip strength, endurance, and the involvement of masticatory and neck muscles can provide valuable insights into the neuromuscular adaptations specific to sport climbing, potentially aiding performance optimization and injury prevention in intermediate and advanced climbers. This study analyzes if the dominant hand has greater isometric endurance and isometric manual grip strength parameters than the non-dominant one and examines its relation to the masticatory and neck muscles in intermediate and advanced sport climbers. *Materials and Methods*: The study was conducted on 41 participants who were divided into two groups according to the IRCRA classification. A level 2 (Lv2) group consisting of 18 climbers and a level 3 (Lv3) group consisting of 23 climbers were identified. First, isometric manual grip strength and isometric endurance were measured using a dynamometer following the protocol of the first three and the last three repetitions (PC3) and the first six and the last six repetitions (PC6). Next, masticatory and neck muscle activity was assessed using surface electromyography, following the same protocol. *Results*: Greater activity of the temporalis muscle was observed on the non-dominant side in the advanced climber’s group (*p* = 0.045). The decrease in bioelectric activity was lower on the dominant side in group Lv3 compared to Lv2. In intermediate climbers, the bioelectrical activity of the masseter muscles in PC3 was greater on the non-dominant side, and in PC6, was greater on the dominant side. The digastric muscles showed a greater decrease in isometric endurance on the non-dominant side. *Conclusions*: Advanced climbers demonstrated greater isometric endurance and isometric manual grip strength parameters in both dominant and non-dominant hands compared to intermediate climbers. The masticatory muscles exhibited higher electromyographic activity on the non-dominant side in the masseter muscles and higher electromyographic activity on the dominant side in the temporalis muscles and digastric muscles.

## 1. Introduction

In recent years, the interest in sport climbing and its role as a way of spending one’s free time has increased significantly. The growing popularity of this sport discipline is associated with its appearance in the program of the Olympic Games, as well as the increasing accessibility to this form of activity [1]. The growing interest in the subject of sport climbing, as well as its competitiveness, have prompted researchers to look for answers to the question of what factors are important in achieving success in this discipline. So far, the existence of numerous anatomical, motor, and training factors has been proven to have a significant impact on achieving high-level climbing results [2]. In sport climbing, the development of upper limb strength and endurance is crucial. This sport is characterized by both sustained isometric contractions and highly coordinated concentric muscle contractions. Isometric work is associated with reduced local blood flow, which leads to the onset of muscle fatigue. Concentric movements require maximized control and stability, while also being performed across a wide range of speeds [3]. Among the key motor elements are the isometric manual grip strength and the isometric endurance of the hand grip, defined as part of the most important determinants of success [2,4]. There are two types of isometric manual grip strength and isometric endurance parameters according to the examination protocol. The static form consists in sustained submaximal grips for a long period, used for a long-term grip hold, and the dynamic form consists of repeated intervals of maximal grips. In the context of sport climbing, the second type of hand grip examination is more adequate to the effort being performed. It corresponds to frequent maximal gripping of the hand in a shorter period while moving along the climbing route [5]. The effort characteristics of sport climbing are often associated with the need to maintain climbing holds and to support the body’s weight on both or on one hand. To increase their climbing abilities, athletes often incorporate in their climbing training special exercises consisting of long-term hanging and pull-ups on a bar or specially designed hang boards [6]. Several scientific reports show that the strength and endurance parameters of the hand grip have a significant impact on the results achieved in sports and can be a necessary factor for reaching a specific level of advancement [7,8]. Previous studies involving athletes have shown that even recreational climbers exhibit greater grip strength compared to athletes from other sports [9]. Additionally, the difference in grip strength between the dominant and the non-dominant hand was smaller among experienced climbers. This may result from the much more frequent involvement of both hands in technically difficult climbing routes, which in many places forces the use of the non-dominant hand, especially as a point of support for the whole body when connecting to subsequent belay points. In contrast, some studies have reported markedly different findings. Some studies have highlighted significant asymmetries in finger flexor strength bilaterally between the strongest and the weakest hand. The dominant hand generated greater strength; however, these results did not correlate in any way with the sporting level or the preferred sport climbing style [10]. The cause of this observation may be the significance of hand dominance influencing the oxygenation kinetics of the flexor digitorum profundus muscle in rock climbers. Research indicates that structural changes in the microvasculature, resulting from the frequent loading of the dominant hand by climbers through isometric contractions, lead to an increase in capillary density and enhanced capillary filtration. They may also be a contributing factor to the higher frequency of injuries observed by researchers in the non-dominant hand [9,11]. In such a specific environment that demands extraordinary precision and stability from the body, often combined with speed, analyzing the parameters of isometric strength and isometric endurance in relation to handedness in athletes is crucial. Due to the significant discrepancies and limited research on this topic in the literature, it is necessary to expand research in this area because of the crucial importance of grip in this sport.

To increase their climbing skills, athletes often incorporate grip strength training into their climbing training. However, studies of athletes from other sports indicate that one mechanism for temporarily increasing various physical parameters is teeth clenching [12,13,14]. This is related to the participation of the masticatory muscles in maintaining the balance of the body by increasing the stability of the head and, at the same time, the line of sight and the body. This is achieved by inhibiting the anti-gravity muscles due to the involvement of the masticatory muscles, which ensure the balance of the head by fixing the temporomandibular joints [15]. The Hoffmann reflex, which is an index indicating the excitation of motoneurons in the spinal cord, is involved in the facilitation of activity in remote muscles associated with voluntary teeth clenching. This facilitation strengthens the masseter muscle activity, making the Jendrassik maneuver more effective, and therefore enhances the activation of remote muscles more effectively [15,16]. As we previously reported, increased functional activity of selected masticatory muscles occurs in sport climbers [17]. This may be related to clenching the teeth during sport climbing activity to generate more strength and endurance in the body’s musculature. It has been proven that a temporary increase in grip strength can be induced by selected oral motor functions, such as teeth clenching [6]. This fact may suggest that sport climbers tend to habitually clench their teeth. However, so far, no studies have examined the relationship between the activity of the masticatory muscles and isometric manual grip strength and isometric endurance parameters.

The aim of this study was to assess the isometric manual grip strength and isometric endurance handgrip parameters, determine the differences in the isometric endurance and isometric manual grip strength of the dominant and non-dominant hands, and evaluate how these relate to masticatory and neck muscle activity in intermediate and advanced sport climbers.

We assumed that the isometric endurance and isometric manual grip strength parameters would be higher in advanced sport climbers compared to intermediate sport climbers, the isometric endurance and isometric manual grip strength parameters would be higher in the dominant hand than in the non-dominant one, and the masticatory and neck muscles on the side of the dominant hand would show greater activity parameters than those on the non-dominant side.

## 2. Materials and Methods

### 2.1. Design of the Study

Before starting the study, the participants were asked to complete an original questionnaire assessing their climbing abilities according to the International Rock Climbing Research Association scale (IRCRA) [18]. Using this scale, the climbers were divided into two groups, i.e., an intermediate group (Lv2, according to the IRCRA scale) and an advanced group (Lv3, according to the IRCRA scale). Then, the participants were familiarized with the study protocol and research equipment. All participants in the study were athletes actively engaged in sport climbing. The grip strength test was carried out using a dynamic protocol consisting of 12 maximal contractions performed on a dynamometer. Following the same procedure, the activity of the masticatory muscles was then tested.

**Figure 1:**
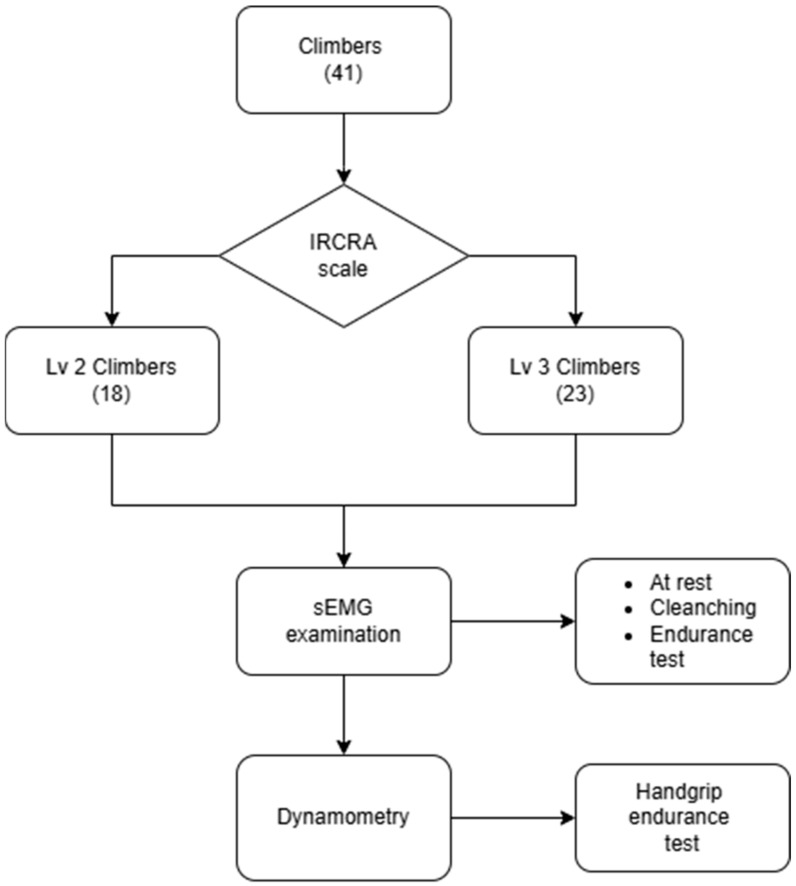
Scheme showing the course of the study.

A total of 71 climbers from various climbing clubs participating in the Academic Polish Championships in sport climbing were invited to take part in the study. After refusals and the application of the exclusion criteria, 41 climbers were included in the study, including 14 women and 27 men. The participants were divided into two groups: 18 sport climbers (6 women, 12 men) of level 2 (Lv2) and 23 sport climbers (8 women, 15 men) of level 3 (Lv3). The participants in group Lv3 were statistically significantly older than the participants in group Lv2 (M = 24.22 vs. M = 22.67, *p* = 0.04) (Figure 1 and Table 1).

The inclusion criteria used in the study were four zones of arch support and complete dentition.

Before qualifying for the study, exclusion criteria were applied and included the following: class II and III malocclusions according to Angle’s classification, orthodontic treatment, open bite, crossbite, inflammatory conditions within the oral cavity, skin diseases affecting the head and neck, neurological disorders (regardless of the type and location), neoplastic diseases (regardless of the type and location), injuries of the head, neck, limbs, and trunk in the last 6 months before the examination, surgical treatment in the last 6 months before the examination (regardless of the type and location), body temperature above 37.5 °C, symptoms of a cold (such as cough or a runny nose), shortness of breath, and sanitary and epidemiological aspects regarding SARS-CoV-2 that did not comply with the current guidelines.

All study participants were informed about the aim of the study and signed an informed consent for participation. The study was conducted in accordance with the standards of the Helsinki Declaration and with the approval of the bioethics committee of the Medical University of Lublin No. KE-0254/113/05/2022.

Initially, a surface electromyography (sEMG) measurement of the masticatory and neck muscles was performed using the 8-K BioPAK measurement system (BioResearch Associates, Inc., Milwaukee, WI, USA). We examined four pairs of muscles in the masticatory and neck muscle groups: the anterior part of the temporal muscles (TA), the superficial masseter muscles (MMs), the anterior belly of digastric muscles (DA), and the sternocleidomastoid muscles (SCMs) [19]. The sEMG measurement was carried out in a sitting position with a leg and head support and was preceded by cleaning the skin of the test area with 90% ethyl alcohol to reduce impedance [19]. The electrodes (Ag/AgCl electrodes with a diameter of 30 mm and a conductive surface of 16 mm—SORIMEX, Torun, Poland) were placed according to the guidelines of surface electromyography for the Non-Invasive Assessment of Muscles program (SENIAM) [20]. Two electrodes were placed on the muscle belly, symmetrically on both sides, following the alignment of the muscle fibers. The sEMG signal was recorded during three conditions: at rest, during clenching, and following the same procedure used to measure the isometric manual grip strength and isometric endurance on a dynamometer. The procedure consisted of 12 maximal teeth clenches on dental cotton rollers. Muscle contraction lasted 4 s with a 2 s break between clenches [21]. The electromyographic signals recorded during the test were amplified as standard and filtered to remove 99% of the noise on a linear scale using the BioPAK digital NoiseBuster filter. Automatic processing of the signals, based on root-mean-square (RMS) calculations in the BioPAK software (version 8.8), provided average values that were subsequently used to analyze muscle activity [19].

Next, the handgrip parameters were tested using the Jamar Plus+ Digital Hand Dynamometer. The study followed the methodology described by Kararanton et al. [21]. The test procedure consisted of 12 maximal hand grips on the dynamometer, held for 4 s, with a 2 s pause between grips. The parameters were selected based on their similarity to movements performed during sport climbing activities.

The parameters used to describe isometric endurance consisted of the percentage change in isometric manual grip strength using the first 3 and the last 3 repetitions [PC = (mean of the last 3 repetitions/mean of the first 3 repetitions) × 100], the percentage change in isometric manual grip strength using the first 6 and the last 6 repetitions [PC = (mean of the first 6 repetitions/mean of the last 6 repetitions) × 100], and the fatigue index [(first repetition − last repetition)/first repetition] × 100] [21,22]. The same equations were applied for the EMG analysis of the masticatory muscles.

The dominance of the hand used was determined by asking the question “which hand do you write with?”. Additionally, to avoid potential errors, the researcher also observed which hand the participant used to complete the questionnaire. Isometric manual grip strength was measured in both dominant (D) and non-dominant (ND) hands. The interval between measurements was set at 15 min to avoid cross-over effects and fatigue [21]. The test was performed first on the dominant hand and then on the non-dominant hand, without informing the subjects of this order. All measurements were taken with the participant sitting, the elbow flexed to 90 degrees and pressed against the torso. The dynamometer spacing was individually set for each subject before testing. The study was conducted with voice commands issued by the researcher. All measurements were performed by a single researcher and recorded using a camera to ensure accurate data transcription and avoid errors.

### 2.2. Statistical Analysis

The normality of the data distribution was examined using the Shapiro–Wilk test. For normally distributed data, parametric tests were used. For each dependent variable, a two-way repeated measures ANOVA was conducted to examine differences based on hand dominance and skill level. Additionally, effect sizes (partial eta squared, η^2^) were calculated to assess the magnitude of the effects. The level of significance was set at *p* < 0.05. For data that were not normally distributed, nonparametric tests were used, i.e., the Mann–Whitney U Test (with continuity correction) and the Wilcoxon matched-pairs test. Cohen’s guidelines for effect size (ES) of Z (nonparametric data) are as follows: a large effect is scored 0.5, a medium effect 0.3, and a small effect 0.1. For partial eta squared (η_p_^2^), η^2^ = 0.01 indicates a small effect, η^2^ = 0.06 indicates a medium effect, and η^2^ = 0.14 indicates a large effect [23].

All statistical analyses were performed using STATISTICA software (ver. 13.1, TIBCO Software Inc., Palo Alto, CA, USA). The results are presented as mean (Mean), standard deviation (SD), median (Mdn), and maximum–minimum ranges. The level of significance was set at *p* < 0.05. This study was considered a “preliminary investigation”; the sample size was not calculated [23].

## 3. Results

### 3.1. Dynamometry

No significant differences in maximal isometric manual grip strength were observed between the dominant and the non-dominant sides or between groups, including comparisons between sides and within each group. There were also no statistically significant differences between groups or dominant and non-dominant sides in the isometric endurance tests. The detailed results of the dynamometry tests are presented in Table 2.

**Figure 2:**
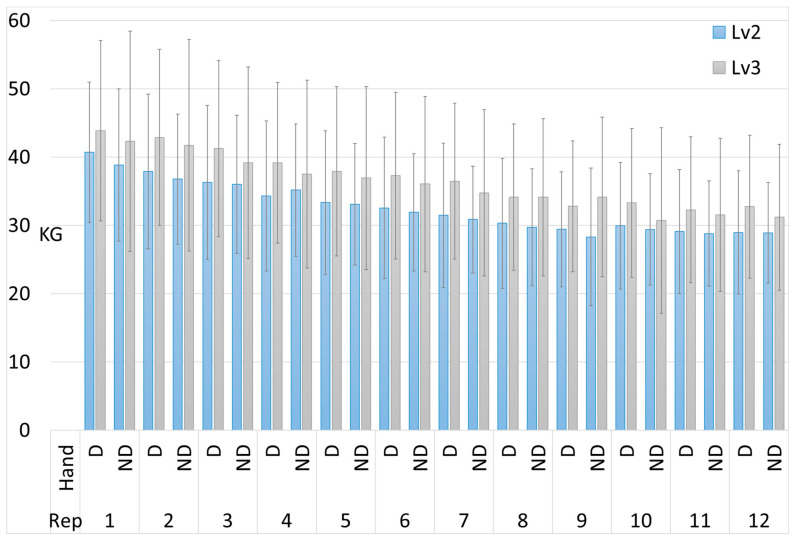
Results of the dynamometry tests for each of the 12 repetitions, comparing the dominant (D) and non-dominant (ND) hand performance in intermediate and advanced climbers. The error bars represent the standard deviation.

The results of the dynamometry tests for each of the 12 repetitions are shown in Figure 2.

### 3.2. Masticatory and Neck Muscle Activity

During teeth clenching, a significant difference was observed between the dominant and the non-dominant sides. Comparisons of the sides within groups reveled that there was a significant difference between the sides only in group Lv3. Greater activity of TA was observed in the non-dominant compared to the dominant side in group Lv3 (M = 223.03 vs. M = 207.92; *p* = 0.045, ES = 0.42, medium effect). There was also a statistically significant interaction in terms of PC3. On the dominant side, PC3 was higher in the Lv3 group than in the Lv2 group (M = 93.47 vs. M = 83.48; *p* = 0.008, ES = 0.17, large effect). No statistical difference was found on the non-dominant side when comparing the groups (Table 3).

For the MMs, a statistically significant effect of the side was observed regarding the PC3 and PC6 indices. PC3 was greater on the non-dominant side than on the dominant one (Lv2: M = 82.07 vs M = 77.67; Lv3: M = 81.93 vs. M = 78.92; *p* = 0.04, ES = 0.12, medium effect). PC6 was greater on the dominant side than on the non-dominant one (Lv2: M = 123.42 vs. M = 119.34; Lv3: M = 120.18 vs. M = 117.10: *p* = 0.04, ES = 0.11, medium effect). It is worth noting that there was also a difference between the sides regarding the FI in favor of the dominant side. However, this result was not statistically significant (*p* = 0.052). The detailed results are presented in Table 4.

Statistically significant differences with medium effect size were found between the preferred and the non-preferred sides in repetition one *(p* = 0.02, ES = 0.36), six (*p* = 0.009, ES = 0.41), nine (*p* = 0.006, ES = 0.43), eleven (*p* = 0.02, ES = 0.37), and twelve (*p* = 0.03, ES = 0.34) in favor of the non-preferred side. Interaction analysis showed that statistically significant side differences occurred in group Lv3 in the first and sixth repetition (*p* = 0.02, *Z* = 0.36; *p* = 0.03, ES = 0.35). In both, the non-preferred side showed greater activity than the preferred one. In group Lv2, statistically greater activity was also observed on the non-preferred side compared to the preferred one in the ninth repetition (*p* = 0.04, ES = 0.32).

There were no statistically significant differences regarding the isometric endurance tests (Table 5).

A significant difference in PC6 between the sides was identified in group Lv2. In this group, greater activity of the DA muscles was observed on the non-dominant side compared to the dominant side (M = 120.27 vs. M = 96.76; *p* = 0.048, ES = 0.52, large effect). There was a statistically significant effect in the group of the FI on the dominant side. The FI was significantly smaller (negative value) in Lv2 compared to group Lv3 (M = −53.85 vs. M = 29.59; *p* = 0.01, ES = 0.44, medium effect). The detailed results are presented in Table 6.

## 4. Discussion

Current research indicates that there are no significant differences in isometric manual grip strength and isometric endurance parameters of the handgrip between advanced and intermediate sport climbers. The research findings on this topic are divided. In contrast to the findings of the presented study, Assmann’s studies demonstrated that climbers had greater grip strength in both hands compared to non-climbers, which is further supported by the results of Grant et al.’s work, in which elite climbers were characterized by much greater grip strength and pinch grip compared to amateur climbers and non-climbers [9,24]. The research results of Giles et al. showed that elite climbers could generate almost twice as much finger grip force as recreational climbers [25]. Similar conclusions were reached by Laffaye et al., whose study found that elite climbers showed higher strength and isometric endurance than skilled climbers and novices [26]. The results from Cutts et al. suggest that sport climbers have greater grip strength and isometric endurance than non-climbers [27]. This research is supported by the work of Vigouroux and Quaine, who compared the grip of elite climbers and non-climbers in terms of strength and the ability to maintain it at high levels [28]. It was proven that elite climbers can maintain a greater finger strength for longer periods than non-climbers. The results of these studies may indicate that the higher the sport climbing level, the greater the isometric manual grip strength and isometric endurance handgrip parameters. However, it should be noted that all the aforementioned studies were based on comparisons between individuals with a high level of climbing proficiency and individuals who were either non-climbers or at a basic climbing level. This represents a significant gap in climbing experience. Our research, on the other hand, compared intermediate climbers to advanced climbers. Chronologically, these levels are closely related, as both groups already practice the sport at least at a moderate level. Therefore, the differences in strength parameters may not be as significant. One of the few studies similar to our work, where intermediate and advanced sport climbers were compared, is the study by Labott et al. [29]. These researchers showed statistically significant differences in grip strength at different elbow positions between intermediate and advanced sport climbers. However, in that study, the skill level of the sport climbers was assessed differently. The international IRCRA scale used in our research was not applied, meaning that the actual skill levels of the participants in that study might not be comparable to the skill levels in our study. The aforementioned studies suggest that the isometric manual grip strength and isometric endurance parameters of the handgrip do not serve as distinguishing determinants between climbers in the Lv2 and Lv3 groups. This is significant from a training perspective, as it highlights the need to focus the training time on developing other skills. Prioritizing abilities that have a greater impact on the climbing performance will be crucial for achieving better results. This provides guidance for coaches on what to emphasize when planning specific training blocks.

In our research, for both groups of advanced and intermediate sport climbers, no statistically significant differences were found in strength and isometric endurance between the dominant and the non-dominant hand. This state of affairs is confirmed by studies conducted by Assmann M., where analyses revealed that experienced climbers were characterized by a smaller discrepancy in grip strength between the dominant and the non-dominant hand than athletes practicing other sport disciplines [9]. This may result from the significantly more frequent use of both hands on technically challenging climbing routes, where the non-dominant hand is often required. Climbers subconsciously treat the non-dominant hand as weaker and may focus more attention on it to increase their abilities. During climbing, the left hand often serves as a stabilizing hand and holds the body weight as a fulcrum, while the right hand is used to attach belay points or connect quickdraws [27]. This results in greater effort and more frequent stimulation of the muscle fibers to increase the strength and endurance of the non-dominant hand. Non-climbers do not experience such frequent stimuli for an isolated stimulation of the non-dominant hand. As a result, the abilities of both hands become more balanced in climbers. Adapting the athlete’s body to the effort he is supported by Laffaye’ s et al. research showing that the type of climbing practiced rather than the level of experience has an impact on climbers’ isometric endurance [26]. Different results were shown in the work of Cutts et al., where sport climbers demonstrated greater isometric endurance in the left hand, which was non-dominant in all participants [27]. However, this difference may be attributed to the use of different measurement tools compared to other studies. It is worth noting that in all studies, the non-dominant hand exhibited the most significant differences in comparisons made with non-climbers. In every case, it was either as strong as or stronger than the dominant hand. This indicates the intensified use of the non-dominant hand in climbing conditions, making it an important element in training. This can be explained by the fact that a less dominant limb tends to have a higher capacity for adaptation than its stronger counterpart, often showing a more pronounced response to training stimuli [30].

The last aim of our work was to assess the relationship between the dominant and non-dominant hand and the isometric strength and isometric endurance of the masticatory and neck muscles. We assumed that the temporal and masseter muscles on the side of the dominant hand would show higher isometric strength and isometric endurance than on the side of the non-dominant hand. We considered the PC3 index that reflects isometric endurance, with higher values indicating a smaller decline in muscle activity during the test and signifying greater isometric endurance. In contrast, the PC6 index represents the inverse relationship, where higher values indicate a greater drop in activity during the test and, thus, lower isometric endurance. The results of our research indicate that, in terms of isometric endurance, there consistently was greater endurance on the non-dominant side for the MMs, accompanied by lower endurance on the dominant side. These findings align with the results obtained for the DA muscles, where endurance was lower on the dominant side. This may stem from the antagonistic nature of these muscles’ functions. The increased endurance and activity on the non-dominant side in the TA muscles could be explained by the previously mentioned phenomenon of equal or greater strength in the non-dominant upper limbs of climbers compared to the dominant ones [9]. The muscles of the cervical spine and the entire region of the neck and upper limbs are related to the masticatory system on many levels, both anatomically and biomechanically. The muscles of the masticatory system, in particular the suprahyoid muscles and infrahyoid muscles along with the mandible, according to the concept of Myers’ myofascial trains, are part of one of the most global anatomical trains [31]. They are an integral part of the deep front train, which, as we pointed out, is responsible for balancing the entire cervical spine and the head positioned on it. This train is involved in all movements of the human body due to its proximity to other myofascial structures. It provides stabilization of the posture of the body’s center and, thus, a stable base for the circumference of the human body, including the upper limbs. Disturbances within the deep anterior train will directly lead to disturbances within the masticatory system. The deep anterior train forms a loop in its cranial course from the inside through the medial pterygoid muscle and outward through the masseter and temporal muscles. In addition, the fascia of the temporalis muscle runs through the skull, heading frontally, and runs in direct contact with the tendon cap, which is the fascia of the scalp, a component of the superficial anterior, posterior superficial, and spiral train, which also cover the muscles of the neck. In addition, the tendon cap lies in direct contact with the suprascapular fascia, which, in the area of the occiput, connects with the nuchal fascia, while it is located superficially and lies near the trapezius muscle that is part of the superficial train of the upper hind limb [32,33,34]. In this way, the concept of anatomical bands explains such an important biomechanical connection of the masticatory system with the neck and the upper limbs [31].

Studies of the masticatory system in athletes of various sports show that teeth clenching can be a mechanism for improving some physical parameters, e.g., balance [15], and is also a form of action of the sympathetic nervous system [35]. It has been suggested that teeth clenching may affect the ability to produce maximum grip force and its speed [6]. So far, only one study evaluated the masticatory muscle tone in sport climbers. In the work of Ginszt et al., it was shown that, compared to non-climbers, climbers were characterized by a higher bioelectrical activity of the masseter muscles during maximal clenching and maximal contraction [17]. It is suggested that by enhancing the Hoffmann reflex, the masseter muscles may improve the activity of the distal muscles [15]. The transfer of tension between the muscles of the masticatory system and the muscles of the upper limbs can also be explained by the existence of myofascial bands [31]. In a study by Danzig et al. in women with temporomandibular disorders who underwent intraarticular injections of the temporomandibular joint, pain in the neck and head decreased, both on the ipsilateral side to the injection and on the opposite side [36].

These findings suggest that training focused on improving isometric endurance and grip strength, particularly for the non-dominant hand, may benefit climbers seeking to improve their performance. Coaches and clinicians could incorporate grip strength exercises with varying intensity levels for intermediate and advanced climbers. Additionally, masticatory muscle conditioning exercises, including controlled clenching, may enhance grip endurance by activating the entire myofascial chain that connects jaw, neck, and upper limbs. This suggests that training focused on isometric endurance and isometric grip strength could be one of the key forms of training that climbers should develop. Given the increased upper body demand in high-difficulty climbing, possessing greater strength and endurance in the arms and shoulders may be advantageous for a better performance [25]. Similarly, Ozimek et al. emphasized the importance of upper body strength and endurance in climbing activities involving high levels of difficulty [37]. Additionally, López-Rivera and González-Badillo underscored the relevance of strength and endurance in climbing [38]. The observed greater bioelectrical activity in the masticatory muscles of climbers may be explained by the principles of tensegrity. Tensegrity is a structural principle in which tension elements (like muscles and tendons) and compression elements (like bones) work together to create a stable, self-supporting structure. This system enables forces to be distributed evenly, allowing the body to maintain stability with minimal energy expenditure [32,39]. This could suggest that tensegrity may be an additional factor worth considering in climbing training.

This research is the first to examine the relationship between the dominant and the non-dominant hand and the ability of the masticatory muscles to generate isometric strength and isometric endurance when clenching the teeth, highlighting the need for more research on this topic. Studies should be conducted in the future involving climbers at other levels of advancement. In relation to the demonstrated interplay between the hand and the masticatory muscles, the incidence of awake bruxism in climbers should also be investigated in the future [35].

This preliminary study has several limitations. First, the small sample size and the lack of a power analysis limit the generalizability of our findings. Future research should consider a larger sample with calculated power to validate these results [23]. Additionally, we did not assess the prevalence of bruxism, which could influence the muscle activation patterns, nor did we measure the bite force, which may also correlate with grip strength in climbers [35]. Including these variables in future studies may provide further insights into the relationship between the masticatory function and upper limb performance in climbers.

## 5. Conclusions

Advanced climbers had greater isometric endurance and isometric manual grip strength parameters in both the dominant and the non-dominant hands than intermediate climbers. The masticatory muscles showed higher electromyographic activity on the non-dominant hand side in the masseter muscles and higher electromyographic activity on the dominant hand side in the temporal muscles and digastric muscles.

It is crucial to understand the differences in physical abilities between climbers of varying skill levels. Characterizing these differences will help design training programs for individuals striving to achieve better performance. Identifying the distinctions between climbers of different proficiency levels will help establish priority training goals, enabling a continuous improvement in performance, both for amateurs and for advanced climbers.

## Figures and Tables

**Table 1:** Anthropometric data.

Group	Weight			Height			Arm Span			Ape Index (cm)			Climbing Experience (Years)		
	Mdn (±SD)	Min	Max	Mdn (±SD)	Min	Max	Mdn (±SD)	Min	Max	Mdn (±SD)	Min	Max	Mdn (±SD)	Min	Max
Lv3	67.70 ± 9.01	45	79	174.50 ± 9.81	152	196	177.76 ± 12.18	149	195	1.017 ± 0.02	0.966	1.067	6.80 ± 4.03	1.75	15
Lv2	65.10 ± 10.45	50	84	172.10 ± 8.11	156	183	176.88 ± 8.74	159	187.5	1.024 ± 0.02	0.983	1.083	3.50 ± 3.98	0.25	13

Lv3—advanced climbers; Lv2—intermediate climbers; Mdn—median; Min—minimum; Max—maximum.

**Table 2:** Results of hand dynamometry tests.

Hand	Lv2			Lv3			Statistics (*p*-Values)			
	Maximal Isometric Manual Grip Strength						Mann–Whitney U Test/Wilcoxon			
	Mean	SD	Mdn	Mean	SD	Mdn	Group	Side	Side Lv3 Int.	Side Lv2 Int.
D [KG]	41.57	10.40	46.70	43.85	13.39	38.40	0.35	0.14	0.14	0.44
ND [KG]	40.97	10.89	44.90	42.54	16.14	42.95	0.89			
	Isometric endurance tests						ANOVA			
	Mean	SD	Mdn	Mean	SD	Mdn	Group	Side	Int.	
D PC3 [%]	76.06	8.13	73.48	76.30	6.54	75.97	0.57	0.46	0.38	
ND PC3 [%]	78.73	7.45	79.05	76.28	8.01	77.25				
D PC6 [%]	120.75	10.27	121.95	120.73	7.56	120.67	0.65	0.60	0.69	
ND PC6 [%]	120.52	8.65	118.14	118.85	8.17	117.71				
D FI [%]	29.28	10.49	30.40	25.95	9.09	26.83	0.51	0.15	0.30	
ND FI [%]	23.95	11.39	26.70	24.70	8.36	26.84				

Lv3—advanced climbers; Lv2—intermediate climbers; D—dominant side; ND—non-dominant side; Int.—interaction; PC3—percentage change in isometric manual grip strength using the last 3 and the first 3 repetitions; PC6—percentage change in isometric manual grip strength using the first 6 and the last 6 repetitions; FI—fatigue index; SD—standard deviation; Mdn—median.

**Table 3:** Results of sEMG of the TA muscles.

Test	Lv2				Lv3			Statistics (*p*-Values) Mann–Whitney U Test/Wilcoxon/ANOVA		
	Hand	Mean	SD	Mdn	Mean	SD	Mdn	Group	Side	Int.
Rest [µV]	D	2.74	2.26	1.99	2.48	§1.90	1.81	0.59	0.90	Lv3 = 0.78
	ND	2.44	1.42	2.47	2.68	2.17	1.90	0.88		Lv2 = 0.62
Clenching [µV]	D	195.86	88.70	185.90	207.91	92.61	210.30	0.57	0.046 * ES = 0.31	Lv3 = 0.045 * ES = 0.42
	ND	203.76	61.10	204.20	223.03	104.10	213.60	0.55		Lv2 = 0.42
PC3 [%]	D	83.48	16.78	80.96	93.47	23.96	88.43	0.45	0.52	0.008 * ES = 0.17
	ND	87.26	25.71	82.60	87.39	19.61	86.07			
PC6 [%]	D	117.11	16.75	116.90	106.73	19.70	105.49	0.2	0.72	0.25
	ND	115.22	24.39	117.12	109.73	20.15	103.44			
FI [%]	D	20.39	19.85	22.00	7.58	26.72	4.85	0.22	0.58	0.20
	ND	18.60	32.70	18.49	11.99	23.58	16.69			

Lv3—advanced climbers; Lv2—intermediate climbers; D—dominant side; ND—non-dominant side; Int.—interaction; PC3—percentage change in isometric strength using the last 3 and the first 3 repetitions; PC6—percentage change in isometric strength using the first 6 and the last 6 repetitions; FI—fatigue index; SD—standard deviation; Mdn—median; *—significant difference.

**Table 4:** Results of sEMG of the MMs.

Test	Lv2				Lv3			Statistics (*p*-Values) Mann–Whitney U Test/Wilcoxon/ANOVA		
	Hand	Mean	SD	Mdn	Mean	SD	Mdn	Group	Side	Int.
Rest [µV]	D	2.02	1.03	1.78	2.90	2.06	2.02	0.31	0.26	Lv3 = 0.11
	ND	2.41	2.49	1.77	2.42	1.44	1.96	0.52		Lv2 = 0.79
Clenching [µV]	D	221.49	116.40	206.10	212.85	126.26	201.70	0.73	0.29	Lv3 = 0.12
	ND	222.48	124.23	209.90	228.88	134.53	204.40	0.86		Lv2 = 0.87
PC3 [%]	D	77.67	18.80	77.51	78.92	18.81	75.75	0.93	0.04 * ES = 0.12	0.68
	ND	82.07	22.14	80.32	81.92	22.90	73.50			
PC6 [%]	D	123.42	18.92	120.43	120.18	22.12	116.17	0.7	0.04 * ES = 0.11	0.77
	ND	119.34	22.55	121.01	117.10	23.31	113.92			
FI [%]	D	30.54	23.74	32.30	25.27	22.15	27.44	0.65	0.05	0.66
	ND	22.84	25.78	30.11	20.34	36.71	34.30			

Lv3—advanced climbers; Lv2—intermediate climbers; D—dominant side; ND—non-dominant side; Int.—interaction; PC-3—percentage change in isometric strength using the last 3 and the first 3 repetitions; PC6—percentage change in isometric strength using the first 6 and the last 6 repetitions; FI—fatigue index; SD—standard deviation; Mdn—median; *—significant difference.

**Table 5:** Results of sEMG of the SCMs.

Test	Lv2				Lv3			Statistics (*p*-Values) Mann–Whitney U Test/Wilcoxon Test			
	Hand	Mean	SD	Mdn	Mean	SD	Mdn	Group	Side Total	Side Lv3	Side Lv2
Rest [µV]	D	1.17	0.32	1.06	1.59	1.50	1.26	0.38	0.48	0.84	0.42
	ND	1.24	0.49	1.13	1.48	0.72	1.36	0.21			
Clenching [µV]	D	16.54	10.88	14.20	18.03	17.41	13.80	0.49	0.10	0.08	0.47
	ND	17.62	9.77	15.95	17.93	9.13	14.80	0.91			
PC3 [%]	D	88.53	39.54	81.03	83.99	26.02	84.07	0.83	0.62	0.21	0.59
	ND	83.69	25.06	79.06	80.91	26.33	81.99	0.87			
PC6 [%]	D	117.79	31.18	117.56	121.39	43.19	109.55	0.85	0.56	0.39	0.88
	ND	120.27	23.69	123.61	122.48	33.99	114.29	0.93			
FI [%]	D	27.35	17.66	25.30	15.40	27.86	12.38	0.18	0.98	0.14	0.09
	ND	20.97	21.52	16.27	19.48	32.25	24.86	0.18			

Lv3—advanced climbers; Lv2—intermediate climbers; D—dominant side; ND—non-dominant side; Int.—interaction; PC3—percentage change in isometric strength using the last 3 and the first 3 repetitions; PC6—percentage change in isometric strength using the first 6 and the last 6 repetitions; FI—fatigue index; SD—standard deviation; Mdn—median.

**Table 6:** Results of sEMG of the DA muscles.

Test	Lv2				Lv3			Statistics (*p*-Values) Mann—Whitney U Test/Wilcoxon Test			
	Hand	Mean	SD	Mdn	Mean	SD	Mdn	Group	Side Total	Side-Lv3	Side-Lv2
Rest [µV]	D	1.56	0.36	1.60	1.72	0.58	1.58	0.49	0.99	0.35	0.27
	ND	1.69	0.69	1.48	1.55	0.43	1.45	0.79			
Clenching [µV]	D	18.58	7.51	17.30	25.93	16.61	22.10	0.28	0.57	0.59	0.08
	ND	22.70	9.61	20.95	25.74	16.35	21.45	0.84			
PC3 [%]	D	176.40	258.70	86.93	80.22	26.20	74.09	0.08	0.25	0.84	0.16
	ND	80.65	18.61	85.03	80.17	24.86	78.46	0.72			
PC6 [%]	D	96.76	34.85	107.99	121.61	27.11	122.61	0.08	0.08	0.71	0.048 * ES = 0.52
	ND	120.27	17.96	115.89	124.90	41.05	122.04	0.99			
FI [%]	D	−53.85	124.16	−3.67	29.59	23.54	32.82	0.01 * ES = 0.44	0.45	0.50	0.10
	ND	3.10	67.71	21.29	25.12	27.52	24.19	0.49			

Lv3—advanced climbers; Lv2—intermediate climbers; D—dominant side; ND—non-dominant side; Int.—interaction; PC3—percentage change in isometric strength using the last 3 and the first 3 repetitions; PC6—percentage change in isometric strength using the first 6 and the last 6 repetitions; FI—fatigue index; SD—standard deviation; Mdn—median; *—significant difference

## Data Availability

The data presented in this study are available on request from the corresponding author. The data are not publicly available due to privacy.
